# Analytical methods and experimental quality in studies targeting carbonyls in electronic cigarette aerosols

**DOI:** 10.3389/fchem.2024.1433626

**Published:** 2024-08-09

**Authors:** Roberto A. Sussman, Federica Maria Sipala, Simone Ronsisvalle, Sebastien Soulet

**Affiliations:** ^1^ Institute of Nuclear Sciences, National Autonomous University of Mexico, Mexico City, Mexico; ^2^ Department of Drug and Health Sciences, University of Catania, Catania, Italy; ^3^ Center of Excellence for the Acceleration of HArm Reduction (CoEHAR), University of Catania, Catania, Italy; ^4^ Ingesciences, Cestas, France

**Keywords:** electronic cigarettes, analytical methods, carbonyls, aerosols, aldehydes

## Abstract

We provide an extensive review of 14 studies (11 independent and three industry-funded) on emissions generated by Electronic Cigarettes (ECs), specifically focusing on the evaluation of carbonyls present in these emissions and emphasizing a meticulous evaluation of their analytical methods and experimental procedures. Since the presence of carbonyl by-products in EC aerosol is concerning, it is important to evaluate the reliability of emission studies quantifying these compounds by verifying their compliance with the following criteria of experimental quality: authors must 1) supply sufficient information on the devices and experimental procedures to allow for potentially reproducing or replicating the experiments, 2) use of appropriate puffing protocols that approach consumer usage as best as possible, 3) use of appropriate analytical methods and 4) usage of blank samples to avoid false positive detection. Outcomes were classified in terms of the fulfilment of these conditions as reliable in seven studies, partially reliable in five studies, and unreliable in two studies. However, only five studies used blank samples and six studies failed the reproducibility criterion. Carbonyl yields were far below their yields in tobacco smoke in all reproducible studies, even in the partially reliable ones, thus supporting the role of ECs (when properly tested and operated) as harm reduction products. This review highlights the necessity to evaluate the quality of laboratory standards in testing EC emissions to achieve an objective assessment of the risk profile of ECs.

## 1 Introduction

Cigarette smoking is responsible for seven million premature deaths each year, including non-smokers passively exposed to exhaled tobacco smoke (Centers for Disease Control and Protection CDC, 2020; World Health Organization WHO, 2023). A global institutional effort has been deployed to address and contain this major health problem, including interventions to prevent smoking initiation and to induce smoking cessation (Athyros et al., 2013; Caponnetto et al., 2013; Kotz et al., 2020). Tobacco Harm Reduction (THR) provides an important and valuable complement to this effort through the substitution of tobacco cigarettes with much safer consumer products such as processed oral smokeless tobacco and Electronic Nicotine Delivery Systems (ENDS) (Amos et al., 2016; Abrams et al., 2018). The latter is a large class of products comprising Electronic Cigarettes (ECs) and heated tobacco products (HTPs), both delivering nicotine through an aerosol generated electronically without combustion. ECs generate an aerosol by condensing vapor produced by heating a liquid solution (the “e-liquid”) with power supplied by an electric battery at temperatures of 180°C–270°C well below tobacco ignition (McNeill et al., 2018; National Academies of Sciences Engineering and Medicine et al., 2018) (HTPs generate a similar aerosol from specially reconstituted tobacco elements). While usage of both types of ENDS is endorsed by several public health experts (Balfour et al., 2021) and has been incorporated in tobacco control policies in the United Kingdom (Office for Health Improvement and Disparities (formerly Public Health England)., 2022) and New Zealand (Ministry of Health NZG, 2024), there are objections to their implementation in public health policies (Ministry of Health NZG, 2024; Pisinger and Døssing, 2014; World Health Organization, 2022), making their usage a controversial issue. However, there is a widespread consensus sustaining that ENDS aerosols expose smokers and bystanders to a significantly reduced level of Hazardous and Potentially Hazardous Compounds (HPHCs) in comparison with cigarette smoke (Amos et al., 2016; Ministry of Health NZG, 2024; U.S. Food and Drug Administration, 2023).

Tobacco smoke is a highly complex set of combustion-originated aerosols, with mainstream emissions inhaled and exhaled by smokers and sidestream emissions emerging from the burning/smouldering tip of cigarettes. Both emissions are generated by the ignition of tobacco biomass at 800°C–950°C, while sidestream emission occurs at 400°C–660°C at the smouldering cigarette tip when the smoker is not puffing (Baker et al., 2004). Environmental tobacco smoke (ETS) is a third aerosol formed by the diluting mixture of mainstream and sidestream emissions in interaction with exogenous environmental pollutants. These emissions expose smokers (and bystanders) to a wide variety of toxicologically relevant HPHCs responsible for multiple adverse health effects (Centers for Disease Control and Prevention et al., 2010). In contrast, aerosols generated by ECs necessarily avoid much of the physicochemical complexity of tobacco smoke, since there is no sidestream emission and their mainstream emission does not contain 97%–99% of compounds (including HPHCs) in tobacco smoke. Emission studies have revealed that carbonyls (specially aldehydes such as formaldehyde, acetaldehyde, and acrolein) are the most abundant (or less negligible) by-products (McNeill et al., 2018; National Academies of Sciences Engineering and Medicine et al., 2018; Office for Health Improvement and Disparities (formerly Public Health England)., 2022), originating from the aerosol formation process from thermal degradation (low-energy pyrolysis or torrefaction) of the ingredients in e-liquids, propylene glycol (PG), and glycerol or vegetable glycerine (VG), which are decomposed into carbonyl compounds, while flavour chemicals also produce by-products (some of which are toxic) from their degradation. Further concerns have been raised for the possible presence of trace levels of metals in the e-liquids and aerosols of ECs, likely leached or transported from the metal components of ECs (McNeill et al., 2018; National Academies of Sciences Engineering and Medicine et al., 2018; Office for Health Improvement and Disparities (formerly Public Health England)., 2022). Carbonyls are particularly concerning because of their association with deleterious health effects. The International Agency for Research on Cancer (IARC) classifies formaldehyde as a human carcinogen (Group 1) (International Agency for Research on Cancer IARC, 2006). Acetaldehyde is possibly carcinogenic to humans (Group 2 B) according to IARC, and acrolein is probably carcinogenic to humans (Group 2A) (Cogliano et al., 2005; International Agency for Research on Cancer IARC, 2021).

To assess health risks in users, quantification of the contents of HPHCs in EC aerosols is essential. This can be achieved by laboratory emission studies in which the devices are puffed with machines simulating user inhalation. Although these tests rely on standardized and regimented puffing protocols that (evidently) do not accurately reproduce real usage, their outcomes might provide the most basic estimation of potential health risks to users. However, given the wide diversity of ECs (devices, coils, e-liquids, nicotine levels, and flavours), there is also a wide diversity of outcomes in the literature on emissions. Hence, to navigate this complexity and to best interpret the objectivity and reliability of these outcomes (and the inferred risk assessment), it is necessary and important to verify (acknowledging limitations) whether the studies comply with basic criteria of experimental quality. Specifically, emission studies must comply with the following requirements: 1) provide sufficient information on the devices and experimental procedures to allow for a potential reproducibility of outcomes; 2) set up appropriate puffing parameters that are as close as possible to the design of the devices and their usage by consumer; 3) use appropriate analytical methods; and 4) use blank samples to control sample contamination. We consider that the degree of fulfilment of these quality criteria is a necessary condition for assessing the reliability of their experimental outcomes.

Two previous review articles (Soulet and Sussman, 2022b; Soulet and Sussman, 2022a) examined 48 emission studies published after 2018 (12 studies on metals and 36 on organic by-products), evaluating the reliability of their outcomes by verifying the fulfilment of the experimental quality requirements listed above. In the present review, we examine 14 studies (Van Leeuwen et al., 2004; Conklin et al., 2018; El Mubarak et al., 2018; Stephens et al., 2019; Gillman et al., 2020; Nicol et al., 2020; Talih et al., 2020a; Rajapaksha et al., 2021; Son and Khlystov, 2021; El-Hellani et al., 2022; Lalonde et al., 2022; McGuigan et al., 2022; Pinto et al., 2022; Talih et al., 2022) not previously considered in (Soulet and Sussman, 2022a), providing a strong emphasis on the detailed critique of the analytical methods. While more than half of the studies reviewed in (Soulet and Sussman, 2022b; 2022a) generated emissions by puffing sub-ohm high-power devices (power settings above 40 W, resistances below 1 Ω) under conditions that favour overheating (see details in (Soulet and Sussman, 2022b; 2022a)), in the present review all 14 studies (except one) examined low-powered devices under appropriate puffing protocols associated with the CORESTA Recommended Method (CRM) 81 (Cooperation Centre for Scientific Research Relative to Tobacco CORESTA, 2015; International Organization for Standardization ISO, 2018c): 3 s puffs, 30 s inter-puff lapse, 55 mL puff volume and 1 L/min airflow rate, or slight variations of these puffing parameters. In addition, most studies used standard appropriate analytical methods, but only six used blank samples, and one-third of the studies (6 out of 14) failed to provide sufficient information to potentially reproduce or replicate the experiments.

This work aims to provide a comprehensive review of emission studies conducted on ECs, with a particular focus on the assessment of the analytical methods employed in measuring carbonyls. Our section-by-section content is as follows. In Section 2, we describe the PRISMA search process for selecting the studies that we revised. Previous reviews are summarized in Section 3, while in Section 4, we present reviews of the 14 studies. A comprehensive discussion and summary of the revised studies are presented in Section 5, and our conclusions are presented in Section 6.

## 2 Methods

**FIGURE 1:**
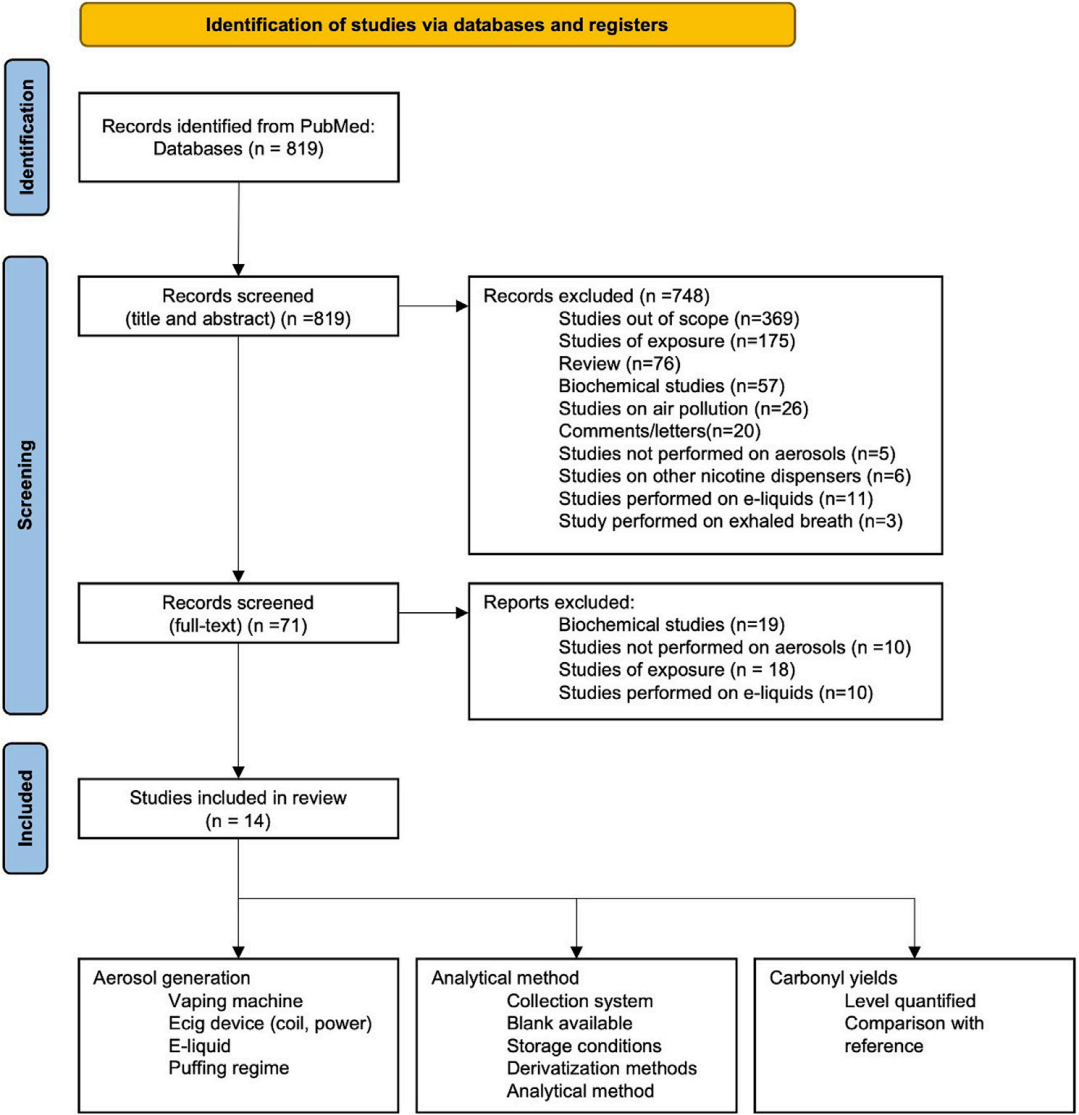
PRISMA-recommended workflow.

We performed a search of the PubMed database of articles on carbonyls in aerosol emissions of conventional cigarettes, electronic cigarettes, and heated tobacco products (see the PRISMA-recommended workflow displayed in Figure 1) (Page et al., 2021). The searched keywords were: {carbonyl OR aldehyde OR formaldehyde OR acetaldehyde OR acrolein} AND {e-cig aerosol OR electronic cigarette aerosol}. The searched terms used to locate articles did not include “HPHCs” or “Toxicants”, which may have resulted in relevant studies that included carbonyls being overlooked.

The initial search was performed on titles and abstracts, excluding articles published before 2018, considering that such studies were revised in the review of carbonyls by Farsalinos and Gillman and published in 2018 (Farsalinos and Gillman, 2018). We also excluded the studies reviewed by Soulet and Sussman (Soulet and Sussman, 2022b). Subsequently, a full-text search was performed by two independent reviewers to exclude articles that did not meet our purposes, such as reviews, exposure studies, biochemical studies, studies not focusing on carbonyls and studies that were not performed on aerosols. No language restriction was applied. We critically analysed the papers that were not excluded to highlight the limitations of the analytical methods, and puffing regimes, and to capture protocols used in the carbonyl analysis of, and ECs aerosols. In particular, we examined the fulfilment of the following criteria of experimental quality:

• Studies conducted on aerosols collected according to the standardized or recommended puffing protocol of the Cooperation Centre for Scientific Research Relative to Tobacco (CORESTA).
• Aerosols were adequately treated for carbonyl entrapment.
• Analytical methods were adequate and reproducible, with particular attention to blank analyses.
• Samples were stored adequately prior to analysis.

The reproducibility criterion follows from demanding that authors provide in their methods section, main manuscript and/or supplementary material, full information of all pertinent parameters, variables and outcomes in their experimental procedures, which makes it possible for other researchers, in principle, to reproduce or replicate the experiments. The following items were assessed to determine reproducibility: operating parameters and characteristics of the devices, e-liquid composition and nicotine concentration, protocols for aerosol generation, sample treatment and analytical method outcomes obtained in all performed repetitions of the experiments that were performed and all data from statistical analyses. If full information was supplied on these items, then the study is considered reproducible (Resnik and Shamoo, 2017; Laraway et al., 2019).

The search resulted in 14 studies, which we review in this article by placing a stronger emphasis on analytical methods, thus providing a more detailed examination of the processes of carbonyl analysis, the derivatization procedure, and the analytical method used for quantitative analysis. The PRISMA-recommended workflow was used (Figure 1).

## 3 Previously published review articles on EC carbonyl emissions

Farsalinos and Gillman published in 2018 (Farsalinos and Gillman, 2018) an outstanding landmark review of 32 studies on the emission of carbonyls from e-cigarette aerosols. Their revision elucidated methodological concerns on laboratory testing of EC emission that were discussed later in (Visser et al., 2021) and in (Soulet and Sussman, 2022a; Soulet and Sussman, 2022b), providing also relevant context for the present review article.

In their discussion section Farsalinos and Gillman provide a detailed description and discussion of the “Dry puff” phenomenon, an organoleptic (sensorially perceived) effect that users of ECs identify with a repellent burning taste in the aerosol. Farsalinos and Guillman explain how this phenomenon occurs when the balance of thermal energy on EC operation is disrupted by supplying excessive power, which produces conditions for a sufficiently rapid e-liquid consumption and its subsequent depletion in the tank, facilitating the pyrolization of the organic material in the wick by the heated coil, hence the users’ perception of a “burning” taste.

While there was already evidence before 2018 that rising supplied power increases aldehyde yields (Geiss et al., 2015; Gillman et al., 2016), Farsalinos and Gillman showed that, besides their organoleptic effect, dry puff conditions prompt a more significant rise of carbonyl yields in the emissions (even surpassing those of cigarette smoke in extreme cases). Farsalinos and Guillman cited and commented previous studies (Farsalinos et al., 2017a; Farsalinos et al., 2017b; Farsalinos et al., 2015; Farsalinos et al., 2018; Geiss et al., 2015) that not only verified users’ recognition of the organoleptic effect of dry puffs (burning repellent taste), but also verified the increase in carbonyl yields by programing their vaping machines to reproduce (as close as possible) the puffing parameters specifically reported by users as associated with a dry puff sensation. The studies cited before (Farsalinos et al., 2017a; Farsalinos et al., 2017b; Farsalinos et al., 2015; Farsalinos et al., 2018; Geiss et al., 2015) were updated more recently by (Visser et al., 2021) in a detailed observational study complemented with analytic quantification of aldehyde levels in the specific puffing parameters that recruited vapers reported as dry puffs sensations.

Farsalinos and Gillman also focused on replications (Farsalinos et al., 2017a; Farsalinos et al., 2017b; Farsalinos et al., 2018) of three studies that had reported excessively high aldehyde yields (surpassing in two of them levels found in tobacco smoke). The replications proved that such excessive yields occurred under dry puff conditions, but aldehydes remained well below their level in tobacco smoke when the devices were tested under lesser power levels in agreement with normal consumer usage.

Since older EC devices lacked power control, users risked being surprised by the sudden emergence of a dry puff, which would evidently cause them to discontinue further puffing, but vaping machines used in the laboratory continue operating. Since only four of the 32 reviewed studies explicitly verified the occurrence of dry puff conditions, Farsalinos and Gillman suggested that several of the remaining 28 reviewed studies might have been conducted without awareness of these conditions, which do not represent normal conditions of realistic consumer usage. This observation prompted them to recommend that emission studies must consider beforehand puffing parameters that avoid these conditions.

The review of 36 emission studies focused on organic by-products by (Soulet and Sussman, 2022b) updated and extended key issues on carbonyls in EC emissions examined by Farsalinos and Gillman 4 years before. Soulet and Sussman also presented an important result previously reported by Talih et al. (Talih et al., 2020b), namely, the existence of a threshold of supplied power that triggers the onset of the exponential increase in the reaction pathways of carbonyl production. Moreover, they provided also a connection between this exponential increase, key puffing parameters (supplied power and airflow rate) and thermodynamical efficiency of the vaping process. They also re-examined the “Dry Puff” phenomenon discussed by Farsalinos and Gillman, showing that this phenomenon might occur along an abrupt process in low-powered devices (as in devices tested before 2018), but might occur gradually in high-power devices whose operational power range is much wider. Soulet and Sussman evaluated the 36 revised studies in terms of quality criteria of experimental design, criteria that we have adopted and adapted to evaluate the 14 emission studies considered in the present review.

## 4 Carbonyls in EC emission studies

In what follows we provide an extensive review of emission studies that have not been previously reviewed. The studies are summarized in Tables 1, 2, and their evaluations are summarized in Table 3.

**TABLE 1:** Summary of analytical methods used in reviewed papers on carbonyls in emissions from e-cigarettes. Information on the usage of blank samples and other method validation is provided in Table 2.

First author	Funding	EC device	Coil and power	Puffing regime	Analytical method	Derivatization method
Conklin et al	Independent	Vaping robot blu cartridges3R4F	1.8 Ω	4 s puff 91 mL puff volume, 2 puffs/min, ISO 3308:2012	GC-MS/FT-ICR-MS	AMAH solution
El Mubarak et al	Independent	EC Nautilus	0.7 Ω 3.7 V, 19 W	30 puffs 3 s inter-puff	UHPLC-UV	DNPH solution
Lee et al	Independent	Not specified	Not specified	10, 20, 30 s puff 10 inter-puff	HPLC-UV	DNPH solution
Stephens et al	Independent	Kanger-tech CE4, EVOD	1.5 Ω	CRM 81 ISO, 20768:2018	CRM 74 ISO 21160:2018	DNPH solution
Gillman et al	Enthalpy Analytical	Innokin iSub	1.2 Ω	CRM 81 ISO 20768:2018	CRM 74 ISO 21160:2018	DNPH solution
Nicol et al	Industry BAT	Vype ePen	Not specified	CRM 81 ISO, 20768:2018 ISO 20778:2018	GS-MS	PFBHA solution
Talih et al	Independent	Juul	1.7 Ω 1.8 Ω	4 s puff, 10 s inter-puff 1 L/min	HPLC-UV	DNPH-cartridges
Rajapaksha et al	Independent	Juul	1.7 Ω 1.8 Ω	CRM 81 ISO, 20768:2018	rtCRDS	No derivatization
Son et al	Independent	ReuLeaux RX200 Aspire Cleito atomizer	50 W, coil resistance not specified	4 s puff 100 mL puff volume, 30 s inter-puff	HPLC-UV	DNPH solution
El-Hellani et al	Independent	Kangertech Subox Mini	Not specified	4 s puff, 10 s inter-puff 8 L/min	HPLC-UV	DNPH-cartridges
Lalonde et al	Industry Juul Labs	Juul	1.8 Ω	CRM 81 ISO, 20768:2018	UHPLC-MS/MS	DNPH solution
McGuigan et al	Independent	Customized vaping machine	Not specified	CRM 81 ISO, 20768:2018	HPLC-MS/MS	PFBHA solution
Pinto et al	Industry BAT	Vype ePod1.0, 1R6F	Not specified	CRM 81 ISO, 20768:2018	GC-MS	DNPH-cartridges
Talih et al	Independent	Juul and 5 disposables	1.64–1.9 Ω	15 puff 4 s puff 1 L/min	HPLC-UV	DNPH-cartridges

**TABLE 2:** Summary of method validation used in reviewed papers. √ represents the presence of analysis blanks. In contrast, x represents the flawed or lack of blank samples.

First author	Method validation	Blank analysis
Conklin et al	*p*-values calculated based on Oneway ANOVA with Tukey adjustment	x
El Mubarak et al	LOD was calculated based on the signal-to-noise ratios of 3 LOQ was calculated using a signal-to-noise ratio of 10	√
Lee et al	The precision of the analytical method was calculated and reported as the mean value ±SD.	x
Stephens et al	FDA guidelines for bioanalytical method validation	x
Gillman et al	LOD was calculated based on the signal-to-noise ratios of 3 LOQ was calculated using a signal-to-noise ratio of 10	x
Nicol et al	LOD was calculated based on the signal-to-noise ratios of 3 LOQ was calculated using a signal-to-noise ratio of 10	√
Talih et al	*p*-values calculated based on Oneway ANOVA with Tukey adjustment	x
Rajapaksha et al	Not provided	x
Son et al	LOD was calculated based on the signal-to-noise ratios of 3 LOQ was calculated using a signal-to-noise ratio of 10	√
El-Hellani et al	The precision of the analytical method was calculated and reported as the mean value ±SD.	x
Lalonde et al	The precision of the analytical method was calculated and reported as the mean value ±SD.	x
McGuigan et al	The precision of the analytical method was calculated and reported as the mean value ±SD.	√
Pinto et al	LOD was calculated based on the signal-to-noise ratios of 3 LOQ was calculated using a signal-to-noise ratio of 10	√
Talih et al	The precision of the analytical method was calculated and reported as the mean value ±SD.	x

**TABLE 3:** Evaluation of the revised studies in terms of fulfilment of the four conditions of experimental quality. We used the symbols √ and x to denote one and zero score points, while “1/2” denotes half a point when a condition was partially fulfilled. Reliability is given in a “traffic light” coloring, with “Reliable” (green) for a score 3.0 and above, “Partially Reliable” (yellow) for a score between 2.0 and 3.0, and “Unreliable” (red) for a score below 2.0.

First author	Provided sufficient information to reproduce results?	Adequate Puffing regime	Adequate Analytical methods	Blanks	Score and Comments
Conklin et al	√	√	√	x	3.0 Reliable
El Mubarak et al	√	√	√	√	4.0 Reliable
Lee et al	Computerize system. Ω NOT disclosed.	?	√	x	1.5 Unreliable
Stephens et al	Ω NOT disclosed	√	√	x	2.5 Partially Reliable
Gillman et al	√	√	√	x	3.0 Reliable
Nicol et al	√	√	√	√	4.0 Reliable
Talih et al	√	Unrealistic puffing regime	√	x	2.5 Partially Reliable
Rajapaksha et al	(1/2) Some results not quantifiable	√	√	x	2.5 Partially Reliable
Son et al	(1/2) Ω of sub-ohm coil not disclosed	(1/2)	√	√	2.0 Partially Reliable
El-Hellani et al	“do-it-yourself” e-liquids, W not disclosed	Unrealistic puffing regime	√	x	1.0 Unreliable
Lalonde et al	√	√	√	x	3.0 Reliable
McGuigan et al	Devices not disclosed	?	√	√	2.0 Partially Reliable
Pinto et al	√	√	√	√	3.0 Reliable
Talih et al	√	√	√	x	3.0 Reliable

Devices and aerosol generation. The authors used a software-controlled (FlexiWare) cigarette-smoking robot (CSR) (SCIREQ; Montreal, CAN), with e-liquids and carrier solutions PG or VG (or mix of PG: VG) from cartridges of commercial blu^®^ EC, loaded into a refillable, clear tank (0.5 mL) atomizer with a coil resistance of 1.8 Ω coupled with a rechargeable blu PLUS + TM (3.7 V) battery (power output 7.6 W) (Conklin et al., 2018). Puffing regime: 4 s puff, 91 mL puff volume, and two puffs/min.

Analytical methods. The authors aimed to study urine biomarkers from whole-body exposure of 12–20-week-old mice to EC aerosols and 3R4F smoke. The levels of carbonyls emitted by the loaded e-liquids with various flavors were quantified. Ten puffs were collected in Tedlar bags. A silicon microreactor coated with 4-(2-aminooxyethyl)-morpholin-4-ium chloride (AMAH) was used to trap carbonyl compounds via oxidation reactions. The analysis was performed using gas chromatography-mass spectrometry (GC-MS) or Fourier transform ion cyclotron resonance mass spectrometry (FT-ICR-MS). Analyses were performed on aerosols from liquids containing only PG, VG, or mixtures at different ratios of 25/75 (PG/VG), 50/50 (PG/VG), and 75/25 (PG/VG). The analysis was carried out by derivatizing the carbonyls with AMAH. This method derivatizes carbonyls by forming oximes using AMAH. The latter must be synthesized, and this can lead to increased error in the reaction with carbonyls if byproducts can be formed in the AMAH synthesis reaction or if it is not purified properly. In addition, before performing GC-MS analysis, the oximes synthesized with carbonyls must be treated with poly-4-vinylpyridine to convert positively charged AMAH adducts to neutral AMA adducts.

Carbonyl yields. formaldehyde 0.25 ± 0.12 μg/puff, acetaldehyde 1.01 ± 0.34 μg/puff, acetone 0.11 ± 0.007 μg/puff, and low levels of crotonaldehyde 0.25 ± 0.12 ×10^−3^ μg/puff were found in the emissions of liquids containing only PG. Acrolein levels were below the detection limit. in VG-only emissions, formaldehyde was 0.59 ± 0.11 μg/puff, acetaldehyde 0.70 ± 0.03 μg/puff, acetone 0.11 ± 0.01 μg/puff and acrolein 0.08 ± 0.002 μg/puff, but crotonaldehyde was below the LOD. The 3R4F cigarette was puffed with 2 s puff, 35 mL puff volume, one puff/min (International Organization for Standardization ISO, 2012). Although the authors made no explicit comparison, aldehydes in EC emissions resulted in significantly lower yields than those in the smoke of the reference cigarette 3R4F.

Device and aerosol generation. A Nautilus atomizer with a 0.7 Ω resistance and supplied voltage of 3.7 V (19.5 W) and a VTC EC were used for sample production (El Mubarak et al., 2018). Thirty puffs were smoked at 3s intervals (El Mubarak et al., 2018).

Analytical methods. This study aims at quantifying the carbonyl compounds in e-cigarette emissions, as they are produced by the decomposition of VG and PG contained in e-liquids. Carbonyls were derivatized with DNPH, by reacting the aerosol sample with a solution of acetonitrile, water, and H_3_PO_4_. Aerosols were collected from the impingers containing a solution of DNPH, water H_3_PO_4_, and acetonitrile. The reaction was run for 30 min, and NaOH was used to alkalize the pH of the solution, allowing it to pass through the chromatography columns. Aerosols were collected from two impingers that contained a solution of DNPH and acetonitrile. Analyses were performed using UHPLC-UV. The method of analysis used is adequate, although the authors state that ultra-performance liquid chromatography coupled with mass spectrometry is more sensitive but still uses UV as the detector.

Carbonyl yields. The results of this study were expressed as carbonyls emitted by e-liquids in μg/puff or μg/mL. In particular, for the carbonyl compounds analyzed, an e-liquid made of 100% PG produced a smaller quantity of carbonyls than that made of 100% VG and the same from e-liquids with VG/PG ratios of 50/50 (PG/VG) and 70/30 (PG/VG). The yields in μg/puff were 0.004 ± 0.0003 (pure PG) and 0.079 ± 0.008 (pure VG) for formaldehyde and 0.011 ± 0.001 (pure PG), 0.053 ± 0.0008 (pure VG) for acetaldehyde.

Device and aerosol generation. The EC device consisted of a mouthpiece, an atomizer to vaporize e-solution at a fixed voltage of 4.2 V, a 2-mL cartridge for storing the EC liquid, and a 900-mAh rechargeable battery. A computerized vaping machine was used with the following puffing protocol: 2 s puff duration and 10 s inter-puff duration, with 5, 10, and 15 puffs in each session. The authors did not specify the percentages of PG and VG contained in the e-liquid, which is particularly relevant because different percentages of these two compounds affect the emission of carbonyl compounds.

Analytical methods. Carbonyl compounds in the EC aerosols were analyzed by derivatization using DNPH and HPLC-UV analysis (Lee et al., 2018). The obtained samples were entrained through an impinger filled with a derivatization solution of acetonitrile and DNPH acidified with H_3_PO_4_, and then analyzed using the HPLC-UV method. It is conceivable that the duration of the reaction is reported for the analysis of carbonyl compounds in e-liquids and, therefore, performed correctly for 30 min. However, it was not possible to precisely determine this, and it was not possible to determine whether the reaction was quenched with a base, as it was not reported by the authors. This could promote secondary condensation reactions and prevent proper quantitative analysis of carbonyls.

Carbonyl yields. Seven carbonyl compounds were analyzed: formaldehyde, acetaldehyde, acrolein, propionaldehyde, butyraldehyde, isovaleraldehyde, and valeraldehyde. In general, formaldehyde was always detected in samples at higher concentrations than those of all other carbonyl compounds. For example, in sample R-2D, the concentration of formaldehyde in μg/mL was 3.2 ± 0.41, while that of acetaldehyde was 0.3 ± 0.01 and acrolein was 0.0002.

Device and aerosol generation. Three generations of EC devices (Kanger-tech CE4, EVOD, and Kanger-tech CE4) and e-liquid formulations with different PV/VG ratios were used. Coils with 1.8 Ω resistance were used for the CE4 and EVOD devices, and power was supplied by an external controllable power supply. The Subox Mini-C includes a 1.5 Ω SSOCC atomizer powered by its battery. Although some characteristics of EC were explained, the author did not state the power setting (in Watts) at which the tests were performed. Aerosols were produced by vaping 50–55 mL puffs for 4 s at intervals of 30 s (CRM 81, ISO 20768:2018) (International Organization for Standardization ISO, 2018c).

Analytical methods. This study aimed to quantify the carbonyls in the emissions from these ECs using a method to trap aerosols consisting of amorphous silica into a syringe for later extraction (Stephens et al., 2019). The aerosols were trapped as droplets on the syringe walls and silica wool threads. The aerosol was recovered from a silica syringe, placed in a centrifuge tube, and centrifuged again. It was then stored at −20°C until analysis. The authors stated that silica wool retained approximately 94 percent of its vaporized liquid mass. Analyses were performed using the CRM 74 method (International Organization for Standardization ISO, 2018a; Cooperation Centre for Scientific Research Relative to Tobacco CORESTA, 2019). The samples were reacted with DNPH for 25 min and then stabilized with Trizma base solution. The derivatized carbonyls were studied using HPLC-DAD and HPLC-MS/MS. Separation was achieved using a C18 column, water, and a mixture of acetonitrile and methanol (1:14) as the mobile phases. The analysis was performed on samples stored at room temperature and −20°C to limit the loss of volatiles. No significant differences were observed among the samples. The recovery of carbonyls by centrifugation was also tested, and the results showed good recovery for all carbonyls, except formaldehyde.

Carbonyl yields. In general, the results for the carbonyls are in agreement with the literature, showing that the use of silica fibers allows for an accurate analysis of carbonyl emissions. In particular, formaldehyde ranged from 0.182 ± 0.023 to 9.896 ± 0.709 μg/puff, and acetaldehyde ranged from 0.059 ± 0.005 to 0.791 ± 0.073 μg/puff. The high upper-end yields of formaldehyde are likely associated with excessive supplied power (a valid assumption since the author did not reveal the power levels used for the aerosol production).

Device and aerosol generation. Aerosols were generated by ten Innokin iSub EC devices (two of which were eliminated from the study) equipped with 1.2 Ω coils powered at 12 W by an Evolv DNA 200 battery unit (Gillman et al., 2020). The devices were tested with four flavored and one non-flavored e-liquids. Puffs were generated by a Cerulean SM450e equipment with the puffing regime of CRM 81 (Cooperation Centre for Scientific Research Relative to Tobacco CORESTA, 2015; International Organization for Standardization ISO, 2018b).

Analytical methods. Highlight differences in carbonyl emission as a function of degradation of the main components of e-liquids, PV, and VG. The unflavoured formulation was homemade to match the PG/VG ratio and nicotine content of the four flavored formulations. The flavored formulations contained a 2/1 ratio of PG/VG and 1.8% nicotine. The unflavored formulation was prepared using a 2/1 ratio of PG/VG and 1.8% nicotine. Tanks and coils were reused for all formulations, emptied, washed with methanol, and air-dried overnight before they were filled with the next formulation. Three replicates were collected for each formulation from 10 ECs, for a total of 30 samples per formulation. Aerosol samples were collected in impingers using a DNPH trapping solution prepared using acetonitrile and H_3_PO_4_, and then quenched with pyridine. The samples were then analyzed using HPLC-UV (Gillman et al., 2016). Separation was achieved using a C18 column and solvent mixture as the mobile phase, as indicated by CRM 74 (Cooperation Centre for Scientific Research Relative to Tobacco CORESTA, 2019; International Organization for Standardization ISO, 2018a).

Carbonyl yields. The flavored formulations tested resulted in a 150%–200% increase in acetaldehyde, no increase or decrease in acrolein, and, depending on the flavored formulation, an increase, decrease, or no change in formaldehyde levels. The methodology of this study was comprehensive and well applied.

Device and aerosol generation. The purpose of this study was to characterize EC emissions using stainless-steel mesh fabric-free distillation plate technology that heats and aerosolizes e-liquid in a single process (Nicol et al., 2020). Carbonyl emissions were compared with those of a reference cigarette, 1R6F, and an EC device EC(BT) Vype ePen. Having ascertained that the device plays an important role, especially regarding the wick and heating coil, the authors developed a novel device that takes advantage of distillation plate technology. IS1.0 (TT) comprises a stainless-steel wire pressed into a mesh structure. It did not contain a wick or a heating coil. According to the authors, this would result in lower levels of carbon emissions. The EC used to compare carbonyl emission levels had the following characteristics: a rechargeable battery section, a replaceable e-liquid-containing cartridge, and a nichrome wire coil heater wrapped around the wick, with a power output of 4.6 W at 3.6 V. The smoking regimen for the reference cigarette, 1R6F, was Health Canada (HCI) (ISO 20778:2018) (International Organization for Standardization ISO, 2018a), while the CRM 81 (ISO 20768:2018) (Cooperation Centre for Scientific Research Relative to Tobacco CORESTA, 2015; International Organization for Standardization ISO, 2018b) was chosen for e-cigarettes.

Analytical methods. The analytical method used for the detection of carbonyls is described in the Supplementary Material. The authors used a non-standardized and unconventional method for the detection of carbonyls. Aerosols collected in the impingers were extracted with water, derivatized with PFBHA, and analyzed using GC-MS. Furthermore, the authors compared the results obtained with the blank sample, which was optimal; however, they used different reference values for IS1.0 (TT) (referred to as IS2.0), 1R6F, and EC(BT): micrograms per 50 puffs, micrograms per cigarette, and micrograms per 100 puffs, respectively.

Carbonyl yields. When the results are compared, it is not clear why the authors maintained different ratios and did not report all the results to one universal unit of measure for all three devices used.

Device and aerosol generation. The authors analyzed the emissions of American and British Juul pods, including their electrical power, total and free nicotine, PG/VG ratio, carbonyls, and reactive oxygen species (Talih et al., 2020a). Liquids and aerosols were analyzed by GC-MS, HPLC, and fluorescence. In particular, aerosols were generated using the AUB Aerosol Lab Vaping Instrument, programmed to perform 15 puffs of 4-s duration, a 10-s interval between puffs, and a flow rate of 1 L/min.

Analytical methods. Carbonyl compounds were trapped on the DNPH cartridges, eluted with 90/10 (v/v) ethanol/acetonitrile, and quantified using HPLC-UV. Gradient elution was performed using a C18 column. The solvents used were water/acetonitrile/THF (60/30/10, v/v/v), water/acetonitrile (20/30, v/v), and acetonitrile.

Carbonyl yields. Compared with the U.S. version, the Juul UK version had approximately one-third the concentration of nicotine in liquids and aerosols. In this case, the authors used an unusual puffing regime, and the final volume of aerosols collected was not reported. Regarding carbonyl compounds, differences were found regarding formaldehyde emissions 4.07 (0.24) (μg)/15 puffs in Juul USA and 3.66 (0.14) in Juul UK, while very similar results were found for acetaldehyde, acetone, and acrolein.

Device and aerosol generation. This paper aimed at analyzing three flavors of Juul pods with 3.7 V and 1.6 Ω coil using a new analytical approach: runtime cavity ringdown spectroscopy (rtCRDS) (Rajapaksha et al., 2021). Aerosols were generated using a peristaltic pump following the ISO 20768:2018 (International Organization for Standardization ISO, 2018d) vaping pattern, but with a higher puff volume to compensate for the relatively weak aerosol generation observed in the Juul device compared with the previous generation devices. The puff duration was 4 s, with a 30 s interval between puffs and a volume of 73.33 mL per puff.

Analytical methods. Aerosols were collected in Tedlar gas bags and analyzed using rtCRDS immediately after aerosol collection. The authors used a fairly novel and highly sensitive technique to identify trace levels of chemicals in air. Specifically, it was used to characterize aerosols with a resolution of a single puff. The technique is based on quantum cascade lasers, which, unlike typical IR, provide low energy to allow the analytes to be excited in their vibrational state, thus allowing for the selective identification of molecules through their molecular fingerprints.

Carbonyl yields. Five spectral datasets were acquired for each sample and the average was used to plot the IR spectra of each sample. The authors claimed that PG oxidation is one of the main sources of acetaldehyde in Juul aerosols. However, the results of the analysis only show the presence or absence of carbonyl analytes; in fact, the analysis is not quantitative, and the results reported are not quantifiable.

Device and aerosol generation. The purpose of this study was to develop a method for producing aerosols from ECs (Son and Khlystov, 2021). The authors developed a vaping machine (E-ACES), analyzed the aerosols produced, and compared them with aerosols produced using conventional smoking machines. An EC ‟mod” type ReuLeaux RX200 and an Aspire Cleito atomizer were used to produce aerosols. The authors used a puff duration of 4 s, vapor volume of 100 mL vaping interval of 30 s, tobacco-flavored e-liquid (30/70 PG/VG), and 6 mg/mL nicotine at 50 W power output. The puffing parameters lead to an airflow rate of 25 mL/s = 1.5 L/min, but coil resistance was not specified, so it is not possible to determine if this airflow was appropriate for a device powered at 50 W.

Analytical methods. Aerosols were passed through glass wool and beads soaked in an acidic solution of DNPH in acetonitrile. Five aerosol puffs were collected and extracted with acetonitrile. For carbonyl analysis using the conventional puffing method, aerosols were passed through DNPH cartridges and extracted with acetonitrile. All samples were analyzed using an HPLC-UV system using acetonitrile and ultrapure water to separate the carbonyl compounds. However, the chromatographic columns used were not described.

Carbonyl yields. The carbonyl levels measured using the DNPH filter/cartridge method and E-ACES were not significantly different, except for benzaldehyde levels determined using the conventional method, which were significantly higher than those determined using the E-ACES method (0.219 ± 0.008 μg/puff vs 0.111 ± 0.026 μg/puff).

Device and aerosol generation. This study analyzed aerosols from “do-it-yourself” e-cigarette liquids (El-Hellani et al., 2022). Aerosols were produced using the AUB Aerosol Lab22 with a puffing regime of 10 puffs for 4s, an interval between puffs of 10s, and a flow rate of 8 L/min.

Analytical methods. A 1 L/min branch was used for carbonyl quantification using DNPH cartridges. There was missing information in this study, which was not found in the Supplementary files. In fact, the characteristics of the ECs are not explained; for example, the coil resistance or supplied power is a significant flaw because this lack of information makes it impossible to reproduce and assess the results of the analysis.

Carbonyl yields. The carbonyl emissions of DIY concentrates, or menthol and tobacco flavorings mixed with DIY additives were comparable to those of commercially flavored e-liquids. The addition of sucralose to PG/VG resulted in a significant decrease in acetone and crotonaldehyde but a significant increase in propionaldehyde. The addition of ethylmaltol resulted in increased acetaldehyde levels. However, these outcomes might vary with alternative “do-it-yourself” e-liquids.

Device and aerosol generation. The authors aimed at testing a “triple puff” method to perform aerosol collection faster and compare it with the traditional method (Lalonde et al., 2022). Juul e-cigs were vaped according to the ISO 20768:2018 regimen (Cooperation Centre for Scientific Research Relative to Tobacco CORESTA, 2015; International Organization for Standardization ISO, 2018b) (55 mL, 3 s, and 30 s between puffs). Single-puff aerosols were produced using linear smoking machines and triple-puff aerosols were produced using rotary smoking machines.

Analytical methods. Carbonyl concentrations were measured by Enthalpy Analytical by Enthalpy’s SOP AM-244. An aliquot of the aerosol condensate was derivatized using DNPH. The analyses were performed by UHPLC-MS/MS. The results of the carbonyl analysis collected using these two methods were then compared with those of the 1R6F reference cigarette.

Carbonyl yields. Acrolein and acetaldehyde were found to be different from the triple puff and single puff methods, which the authors attributed to the degradation of acetaldehyde. To achieve these considerations, the authors failed to accurately describe the analytical method, which rendered it impossible to reproduce (a serious flaw).

Device and aerosol generation. The purpose of this study was to develop a quantitative method for measuring four harmful carbonyls (acetaldehyde, acrolein, crotonaldehyde, and formaldehyde) in aerosols generated by ECs (McGuigan et al., 2022). Aerosols were formed using a CETI-8 vaping machine following the CRM 81 method (ISO 20768:2018) (Cooperation Centre for Scientific Research Relative to Tobacco CORESTA, 2015; International Organization for Standardization ISO, 2018b). The authors did not provide a description of EC devices.

Analytical methods. The tested method used a commercially available sorbent bed treated with a derivatization solution to trap and stabilize the carbonyls. Analytes were extracted from the sorbent material using acetonitrile and analyzed using HPLC-MS/MS. Separation was performed using a C18 column equipped with a precolumn. Water with ammonium acetate and acetonitrile were used as the mobile phase.

Carbonyl yields. The devices produced aerosols containing the following ranges of carbonyls: acetaldehyde (0.0856–5.59 μg), acrolein (0.00646–1.05 μg), crotonaldehyde (0.00168–0.108 μg) and formaldehyde (0.0533–12.6 μg). The study reported that the method blank samples collected and analyzed daily showed no residual carbonyl content in solvents, cartridges, or vaping. The authors did not describe the EC devices used for aerosol generation, which is a serious shortcoming that prevents assessing the study outcomes on the formation of carbonyl byproducts.

Device and aerosol generation. In this study, aerosols emitted from fourth generation “pod” EC devices Vype ePod1.0 using microporous ceramic as the wicking material were compared with those emitted from the conventional reference 1R6F cigarette (Pinto et al., 2022). The EC operates within a range from non-adjustable 2.2–3.1 V non-adjustable, a power of 6.5 W, resistance NiCr, 0.8–1.4 Ω. Conventional cigarettes were smoked according to the ISO intense smoking regime typically using 9–10 puffs. EC aerosols were generated according to ISO 20768:2018 (International Organization for Standardization ISO, 2018d) using a rotary or linear puffing machine. Cigarette and EC emissions were sampled and analyzed in five independent replicates.

Analytical methods. Determination of selected carbonyls in E-liquids, EC aerosols, and mainstream tobacco smoke using PFBHA derivatization and gas chromatography/mass spectrometry analysis. The Pads were extracted using an acetonitrile impinger solution. A portion was diluted with Type I water and derivatized with PFBHA, followed by extraction in toluene. The samples were analyzed using GC-MS.

Carbonyl yields. The results showed that carbonyl levels were significantly reduced compared with those in other studies on e-cigarettes and 1R6F cigarettes.

Device and aerosol generation. The purpose of this study was to analyze aerosol emissions from various disposable ECs and compare them with those from Juul devices (Talih et al., 2022). The vaping pattern was performed with the AUB Aerosol Lab Vaping Instrument consisting of 15 puffs for 4 s at a flow rate of 1 L/min.

Analytical methods. The aerosols were passed through a DNPH cartridge, washed with a 90/10 (v/v) ethanol/acetonitrile solution, and quantified using ultraviolet high-performance liquid chromatography (HPLC-UV). Gradient elution was performed using a C18 column. The solvents used were water/acetonitrile/THF (60/30/10, v/v/v), water/acetonitrile (20/30, v/v), and acetonitrile.

Carbonyl yields. Except for the SEA device, the single-use products generated significantly more toxicants than the Juul.

## 5 Discussion

Low-molecular-weight carbonyl compounds such as acetaldehyde, acrolein, and formaldehyde are among the most toxic compounds in cigarette smoke. The main sources of these aldehydes are carbohydrates found naturally in tobacco or in the components of e-liquids that undergo thermal degradation, resulting in the emission of carbonyl compounds (Baker et al., 2005).

Consequently, the amount of these aldehydes in the emissions generated by various tobacco products needs to be examined and determined to best assess the potential toxicity of these compounds in EC aerosol. In the 14 studies considered in the present review the detected levels of the main aldehydes (formaldehyde, acetaldehyde and acrolein) cluster below 1 mg/puff. These are considerably lower values than the levels of same compounds found in the smoke of cigarettes used in laboratory tests (Counts et al., 2005): 7.5–12.5 mg/puff (formaldehyde), 50–150 mg/puff (acetaldehyde), 7.5–15 mg/puff (acrolein), where we assume 10 puffs per cigarette to obtain these values.

### 5.1 Derivatization methods

Several analytical methods for the derivatization of carbonyl compounds are described in this review. However, most studies, particularly 10 papers, reported the use of 2,4-DNPH for the formation of hydrazones, that is, adducts between carbonyl compounds and DNPH (Cox et al., 2016), as recommended by CRM 74 (Cooperation Centre for Scientific Research Relative to Tobacco CORESTA, 2019; International Organization for Standardization ISO, 2018a). The reaction of a carbonyl compound with DNPH is an addition-elimination reaction catalyzed by an acidic environment. The acid activates the carbonyl group via the formation of a carbocation. This is more easily attacked by a nucleophile consisting of the amine of DNPH through nucleophilic addition. A tetrahedral intermediate was then formed. The elimination of water then occurs, resulting in the formation of 2,4-dinitrophenyl hydrazone. The pH of the solution should be adequately controlled to prevent excessively acidic conditions that could lead to the condensation of carbonyl compounds and the consequent inability to quantify them. Furthermore, the optimal reaction time for carbonyl compounds with DNPH was estimated to be 30 min, which allowed the derivatization of all carbonyls in the solution and prevented the formation of polyderivative compounds. The reaction is usually quenched with a pyridine solution to basify the solution and prevent polyderivatization reactions. In particular, six studies reported the use of an acidic solution of DNPH in acetonitrile, and four used a DNPH cartridge or DNPH silica. Compared to impingers containing an acidic solution of DNPH in acetonitrile, DNPH cartridges exhibit limitations. The first can be saturation, which could occur during the same analysis and may also depend on the volume of aerosol passing through the cartridge. Condensates can be deposited on cartridges, which can impair their ability to retain carbonyls. Additionally, oxidants in the air may cause secondary reactions with DNPH and interfere with the study of carbonyls (Uchiyama et al., 2011). Furthermore, with unsaturated carbonyls (such as acrolein), the possibility of the formation of polymerization byproducts has been demonstrated, preventing the proper analytical identification of unsaturated carbonyl compounds (Van Leeuwen et al., 2004).

Two studies (Nicol et al., 2020; McGuigan et al., 2022) used the PFBHA derivatization technique. Derivatization with PFBHA is the second most widely used reaction following derivatization with DNPH (Szulejko and Kim, 2015). The reaction occurs through nucleophilic addition of PFBHA to the carbonyl, with the formation of an intermediate. Finally, following the removal of a water molecule, a derived oxime was formed (Bao et al., 2014). Conklin et al. used a derivatization reaction with a silicon microreactor coated with 4-(2-aminooxyethyl)-morpholin-4-ium chloride AMAH (Conklin et al., 2018). Rajapaksha et al. collected the aerosols in a Tedlar bag and analyzed them directly in a gas chromatograph coupled to a mass spectrometer and rtCRDS, without derivatizing them (Rajapaksha et al., 2021). The numerous derivatization methods employed pose a challenge in making direct comparisons between study values owing to the diverse parameters utilized.

### 5.2 Analytical methods

Although several analytical methods have been reported for the quantification of carbonyls, eight studies have used the HPLC-UV method. The HPLC-UV method is the most widely recognized method recommended by CORESTA, although the protocol recommended by CORESTA was not followed by all the authors. The CORESTA recommended methods for HPLC analysis are CRM 74 and CRM 96. CRM 74 outlines the procedures for quantifying all carbonyls in conventional cigarette smoke through derivatization with DNPH and subsequent HPLC-UV or HPLC-DAD analysis (International Organization for Standardization ISO, 2018a). On the other hand, CRM 96 describes procedures for quantifying formaldehyde and acetaldehyde in e-cigarette aerosols using the same methods of derivatization with DNPH and HPLC-UV or HPLC-DAD analysis (Cooperation Centre for Scientific Research Relative to Tobacco, 2024).

In the reviewed studies, chromatographic separation was performed using a reversed-phase C18 column as the stationary phase. Notably, reversed-phase columns retain less polar compounds than more polar compounds. The use of a C18 column increases selectivity because of its high surface coverage. In addition, gradient separation was performed in all the studies using liquid chromatography. This is because the analytes have different hydrophobicities, and gradient elution allows for rapid analysis. Moreover, all studies that used a UV spectrometer as a detector used a wavelength of 360 nm, which prevents the detection of extraneous peaks with higher absorbance at shorter wavelengths. Despite the widespread use of liquid chromatography coupled with ultraviolet spectrophotometry, there are some issues related to the fact that this methodology was developed for conventional cigarettes and then applied to ECs. Therefore, it would be more appropriate to apply CRM 96, although it outlines operational procedures for the analysis of formaldehyde and acetaldehyde only. Flavors contained in e-liquids have been shown to cause analytical interference in the HPLC-UV method. In addition, mass spectrometry coupled with liquid chromatography (LC-MS) is an analytical technique with better selectivity and sensitivity. This technique, applied to the study of aerosols from ENDS, allows a more accurate quantification of low-concentration carbonyls (mainly medium- and high-molecular-weight carbonyls) and more adequately quantifies these molecules (Flora et al., 2017; Zhu et al., 2020).

Finally, one technique that was not identified in the studies was Gas Chromatography coupled with two-dimensional separation (GCxGC) and various detection methods like Time-of-Flight Mass Spectrometry (TOFMS) and Flame Ionization Detection (FID). Despite its limited use in detecting carbonyls, this technique is considered valuable for identifying volatile organic compounds (VOCs) because of its enhanced selectivity.

In addition to capture and analysis techniques, the composition of e-liquids must also be well-characterized, as they can affect the emission of carbonyls (Qu et al., 2018). The PV/VG ratio and the presence of flavoring agents influence the production of carbonyls. In fact, all the studies examined in this review have well-characterized the characteristics of e-liquids before performing the analysis (Van Leeuwen et al., 2004; Uchiyama et al., 2011).

### 5.3 Blank analysis and sample storage

Only five of the 14 revised studies used blank samples (Table 2). The blank method involves the analysis of an analyte-free matrix processed using the same method as that used for the analysis. Blank analysis plays a key role in quantitative investigation because it excludes contamination from the results of the analysis. Among the studies reviewed, only six performed or presented a blank analysis of the air. The handling of blank samples is a critical component of chemical analysis. It is essential to closely monitor the results of blank samples during the analysis process to identify anomalies that may suggest issues with collection or analysis procedures. Furthermore, it is crucial to incorporate the results of blank samples into reports or publications to ensure transparency and enable others to comprehend the data more effectively. The results of the blank samples should be presented clearly and concisely alongside those of the other samples. It is important to verify whether the data are derived only from the sample and not from contamination outside the sample (Cantwell, 2019).

This represents a serious shortcoming in the accuracy, treatment, and interpretation of the analyzed data. Table 2 illustrates that the above-mentioned studies utilized a range of alternative methods to validate the analytical techniques in question. Generally, the limits of detection and quantification are determined by considering the signal in relation to the background noise. Other studies have employed varying numbers of analyses and have reported results in terms of standard deviation.

Furthermore, almost none of the reviewed studies reported how the samples, e-liquids and devices were stored before the analysis. To understand and reproduce these results, it is necessary to know how the devices were maintained, to rule out any form of instrument spoilage or deterioration, and whether the e-liquids of electronic cigarettes were maintained under appropriate temperature and light conditions to exclude the formation of internal secondary reactions. According to the guidelines outlined in the CRM Method 18, devices and aerosols are recommended to be stored at room temperature. This applies specifically to e-liquids, which should be kept in hermetic containers to prevent the accidental ingestion of water. These conditions are necessary when devices and aerosols are used within a short period of aerosol production. However, if these conditions are not applicable, both e-liquids and cartomizers should be stored at a temperature of at least −10 °C. Furthermore, Jităreanu et al. (2022) recently showed that the storage period of e-liquids, storage temperature, and type of cartomizer can strongly influence the concentrations of metals within the liquids (Jităreanu et al., 2022). In addition, for studies comparing the emissions of ECs with those of conventional cigarettes, it is necessary to determine whether the latter has been maintained at an optimal level of temperature and humidity. Similarly, the atmospheric conditions during the analysis are significant for ruling out any kind of analytical interference. Moreover, it is important to know the storage conditions of the collected aerosol samples if they are not analyzed immediately after collection.

### 5.4 Puffing regimes

The puffing regime in laboratory testing strongly influences the production of carbonyls from e-cigarette emissions. A puffing regime is characterized by four basic parameters: puff duration, puff volume, intervals between puffs, and the airflow rate. There is evidence that variation in even one of these parameters can change the quantity of carbonyls emitted (Bao et al., 2014; Cooperation Centre for Scientific Research Relative to Tobacco CORESTA, 2015). Laboratory emission studies are essential to evaluate quality control and compare EC devices, hence vaping machines require to puff the devices with a protocol of regimented puffs which should (ideally) be systematized within a recognized standard. So far, most emission studies rely on the ISO 20768:2018 CORESTA standard (Cooperation Centre for Scientific Research Relative to Tobacco CORESTA, 2015; International Organization for Standardization ISO, 2018b) (3 s puff, 30 s inter-puff, 55 mL puff volume, airflow 1.1 L/min), or minor variation of these parameters. Evidently, no regimented puffing will reproduce inhalation patterns of the widely varying patterns of consumer usage, but puffing protocols can provide a reasonable proxy of human exposure if they approximate as best as possible consumer puffing patterns for specific devices and vaping styles. In particular, the puffing parameters of the ISO 20768:2018 CORESTA standard were conceived to test low-powered devices, mostly manufactured by the tobacco industry; hence, they provide a reasonable proxy approximation to puffing patterns of generic low-powered devices (<20 W) used for the mouth-to-lung style (see Section 3). In the present review (see Sections 6 and 7), 24 of the 30 revised studies tested low-powered devices, mostly cartridge-based pods, using the CORESTA standard or minor variations, which are adequate for these devices.

The reviews by Soulet and Sussman, examined several studies in which emissions were generated by puffing various brands of high-powered sub-ohm devices with the CORESTA protocol (or close deviations of it). This combination of high supplied power and low airflow favors overheating conditions that might lead in extreme cases to a dry puff. However, these conditions do not apply to the studies examined in the present review and will not be discussed any further (for details on the problems found in high powered devices see (Soulet and Sussman, 2022b; Soulet and Sussman, 2022a)).

### 5.5 Evaluation of the studies

In Table 3, we display our evaluation of the reliability of the 14 revised studies in terms of fulfilment of the four criteria of experimental quality: reproducibility, adequacy of puffing regime and analytical methods, and usage of blank samples. We used a “traffic light” coloring of scores, with “Reliable” (green) for scores 3.0 and above, “Partially Reliable” (yellow) for scores between 2.0 and 3.0, and “Unreliable” (red) for scores below 2.0. We assigned a score of 1/2 to studies that partially complied with a given condition or studies that tested both low and high-powered devices, with the latter ones with inappropriate airflow.

Table 3 shows seven studies that are “Reliable”, 5 “Partially Reliable” and 2 “Unreliable”. It is interesting to see cross references between the different quality conditions. Surprisingly, only one of the 7 Reliable studies used blank samples (only three studies used blank samples). Six studies failed the reproducibility criterion. As described in the reviews of Section 4, practically all studies used reasonable values of puff duration (around 3s) and inter-puff intervals (30s and 60s).

## 6 Conclusion

Since the introduction of ECs into global markets as safer substitutes for tobacco cigarettes, hundreds of studies have been undertaken to examine the chemical contents of their aerosol emissions as an essential part of the process to fully understand and evaluate their toxicity and risk profile, a process whose outcomes are crucial to inform and guide all stakeholders (consumers, health professionals, regulators, and industries).

Emission studies are the ground-level stage in the evaluation of the risk profile of ECs, followed by preclinical and clinical studies. Reviews of these studies fulfil the important task of collecting and revising the “state of the art” research in each topic, but to be useful they must go beyond merely citing the studies and listing their outcomes; they must also provide detailed and critical assessments of the involved methodology and the consistency of experimental outcomes. With this purpose in mind and considering that carbonyls (especially aldehydes) are the most frequent and abundant toxic byproducts found in EC emissions, we have presented in this review a detailed and critical examination of the experimental procedures, analytical methods, and outcomes of 14 recently published emission studies focusing on carbonyls (the studies are summarized in Tables 1 and 2). Our review complements two of our own recently published reviews of emission studies targeting toxic byproducts (metals (Soulet and Sussman, 2022a) or organic byproducts (Soulet and Sussman, 2022b)) published after 2018 (we omitted studies published before 2018, as they tested devices that are currently obsolete or of marginal use).

To evaluate the reliability of the studies in our review we examined compliance with (what we regard) as the minimal requirement of experimental quality stated in Section 2, namely, authors must provide: 1) sufficient information on the devices and on all experimental procedures to be reproducible, 2) use of appropriate puffing parameters to generate aerosols, 3) use appropriate analytic methods and 4) use blank samples. From the detailed reviews in Section 4 we defined a gradation system in terms of a “traffic light” point classification given in terms of their degree of compliance with the four quality conditions. The details of this evaluation are displayed in Table 3, showing that seven studies were Reliable, 5 Partially Reliable and 2 Unreliable. Most studies used appropriate analytical methods (although some failed to provide sufficient information on various issues), and most obtained aldehyde yields that are negligible or well below yields in cigarette smoke. However, the studies exhibited the following experimental flaws:

• Although only five of the 14 studies used blank samples, alternative validation methods have been employed.
• six studies failed the replicability condition by not disclosing sufficient information on the devices and experimental procedures.

The issues listed above are serious methodological flaws that occur also in many emission studies (see (Soulet and Sussman, 2022a; Soulet and Sussman, 2022b)). Evidently, these flaws need to be corrected to improve the quality of emission testing, which suggests the need to update and improve the standards of laboratory emissions testing. Testing standards (as well as the peer review process evaluating emission studies) must incorporate and demand the usage of blank samples and that authors supply all relevant information for potentially reproducing or replicating the experiments. In addition, testing standards must overcome the rigidity of considering only the CORESTA airflows (or minor variations) for testing all devices, including high-powered ones used for Direct to Lung style (see criticism on this point in (Soulet and Sussman, 2022a; Soulet and Sussman, 2022b)). As we have suggested in previous reviews, it is also important to incorporate EC users within the experimental logistics, since after all the devices are aimed for consumers. Unfortunately, many emission studies simply ignore the peculiarities of consumer patterns.

Updating and improving testing standards to incorporate basic conditions of experimental quality is necessary to achieve a more objective evaluation of the risk profile of ECs, which will provide valuable information to all stakeholders (consumers, health professionals, regulators, and the industries themselves). We will continue to review emission studies, and in future research we will conduct our own laboratory studies based on the quality conditions we have indicated in our literature review.

## Figures and Tables

**Table:** 

**AMAH**	4-(2-aminooxyethyl)-morpholin-4-ium chloride
**CO**	Carbon Monoxide
**CORESTA**	Cooperation Centre for Scientific Research Relative to Tobacco
**CRM**	CORESTA Recommended Method
**CSR**	cigarette-smoking robot
**DIY**	Do It Yourself
**DNPH**	2,4-Dinitrophenylhydrazine
**DTL**	Direct To Lung
**EC**	Electronic Cigarette
**ENDS**	Electronic Nicotine Delivery Systems
**ETS**	Environmental tobacco smoke
**FDA US**	Food and Drug Administration
**FID**	Flame Ionization Detection
**GCxGC**	Gas Chromatography x Gas Chromatography
**GC-MS**	Gas Chromatography–Mass Spectrometry
**FT-ICR-MS**	Fourier transformation cyclotron resonance mass spectrometry
**HCI**	Health Canada Intense
**HPHC**	Hazardous and Potentially Hazardous Compounds
**HPLC-DAD**	High-Performance Liquid Chromatography Diode Array Detector
**HPLC-MS/MS**	
**HPLC-UV**	High-Performance Liquid Chromatography Ultraviolet
**HTP**	Heated Tobacco Products
**ISO**	20778:2018 CORESTA FTC puffing regime (2 s puff 60 s inter-puff 35 mL puff volume)
**IARC**	International Agency for Research on Cancer
**IR**	infrared spectroscopy
**ISO**	20778:2018 HCI puffing regime (2 s puff 30 s inter-puff 55 mL puff volume)
**ISO**	International Organization for Standardization
**LOD**	Limit Of Detection
**LOQ**	Limit Of Quantitation
**MEV**	mass of e-liquid vaporized
**NaOH**	Sodium hydroxide
**PG**	Propylene Glycol
**rtCRDS**	runtime cavity ringdown spectroscopy
**SD**	Standard Deviation
**PFBHA**	o-(2,3,4,5,6-Pentafluorobenzyl)hydroxylamine
**THF**	Tetrahydrofuran
**TOFMS**	Time of Flight Mass Spectrometry
**UV**	Ultraviolet
**UHPLC-MS/MS**	Ultra-High-Performance Liquid Chromatography Mass Spectrometry
**UHPLC-UV**	Ultra-High-Performance Liquid Chromatography Ultraviolet
**VG**	Vegetable Glycerin or Glycerol
**WHO**	World Health Organization
